# Laparoscopic Modified One Anastomosis Gastric Bypass for Severe Obesity After Pancreaticoduodenectomy: A Safe Approach to the Hostile Abdomen, a First Case Report

**DOI:** 10.1002/kjm2.70233

**Published:** 2026-05-07

**Authors:** Chih‐Kun Huang, Ming‐Che Hsin, Long‐Bin Jeng, Ting‐Wei Chang

**Affiliations:** ^1^ College of Medicine, China Medical University Taichung City Taiwan; ^2^ Division of Metabolic and Bariatric Surgery, Department of Surgery China Medical University Hospital Taichung City Taiwan; ^3^ Body Science and Metabolic Disorders International Medical Center, China Medical University Hospital Taichung City Taiwan; ^4^ Organ Transplantation Center, China Medical University Hospital Taichung Taiwan; ^5^ Department of Surgery China Medical University Hospital Taichung Taiwan; ^6^ Cell Therapy Center, China Medical University Hospital Taichung Taiwan; ^7^ Weight Management Center, Kaohsiung Medical University Hospital/Kaohsiung Medical University Kaohsiung City Taiwan; ^8^ Division of Thoracic Surgery, Department of Surgery Kaohsiung Medical University Hospital/Kaohsiung Medical University Kaohsiung City Taiwan

1

Long‐term survival after pancreaticoduodenectomy (PD) has improved significantly, leading to an increasing number of post‐PD patients presenting with metabolic syndrome and severe obesity [1]. However, performing metabolic surgery in this population presents a formidable technical challenge. The “hostile abdomen,” characterized by dense adhesions and complex, altered vascular and intestinal anatomy, makes standard procedures hazardous [2]. Re‐entering the right upper quadrant to perform a surgical procedure has risks of injuring the precarious pancreaticojejunostomy or choledochojejunostomy, potentially leading to catastrophic leaks or fistulas [2, 3]. To address this dilemma, we adopted a “safety‐first” strategy that provides metabolic efficacy without disturbing the original critical reconstruction area. We describe the technical details and outcomes of laparoscopic modified one anastomosis gastric bypass (mOAGB) in a patient with morbid obesity following a conventional Whipple operation.

A 42‐year‐old female presented with severe obesity 5 years after undergoing a standard Whipple procedure for a benign pancreatic head tumor. Despite the previous surgery, she developed severe obesity with a body mass index (BMI) of 42.6 kg/m^2^ (113.2 kg). Her comorbidities included essential hypertension, type 2 diabetes mellitus, and hyperlipidemia. Preoperative evaluation, including abdominal ultrasonography and upper gastrointestinal endoscopy, confirmed a patent gastrojejunostomy (GJ) without marginal ulceration and ruled out tumor recurrence. Because of her severe obesity and multiple metabolic comorbidities, a purely restrictive procedure was considered potentially insufficient for durable metabolic control. However, extensive revisional bypass involving dissection around the pancreaticojejunostomy or choledochojejunostomy was considered high‐risk in this hostile post‐Whipple abdomen. Therefore, we selected a hybrid strategy combining gastric restriction with limited intestinal rerouting to achieve both restrictive and bypass‐related metabolic effects while preserving surgical safety. The patient was placed in the supine reverse Trendelenburg position under general anesthesia. A four‐port laparoscopic approach was utilized. Upon entry, dense adhesions were encountered throughout the supramesocolic compartment. Meticulous sharp dissection was performed to expose the hiatus and the anterior gastric wall. Crucially, our dissection strategy was strictly confined to the inframesocolic and gastric regions. We deliberately avoided the hepatoduodenal ligament area to protect the previous pancreatic and biliary anastomoses. The previous GJ with a Braun enteroenterostomy was identified. The afferent limb measured 20 cm from the biliopancreatic entry to the Braun anastomosis, and the segment from the GJ to the Braun measured 10 cm (Figure 1A).

**FIGURE 1:**
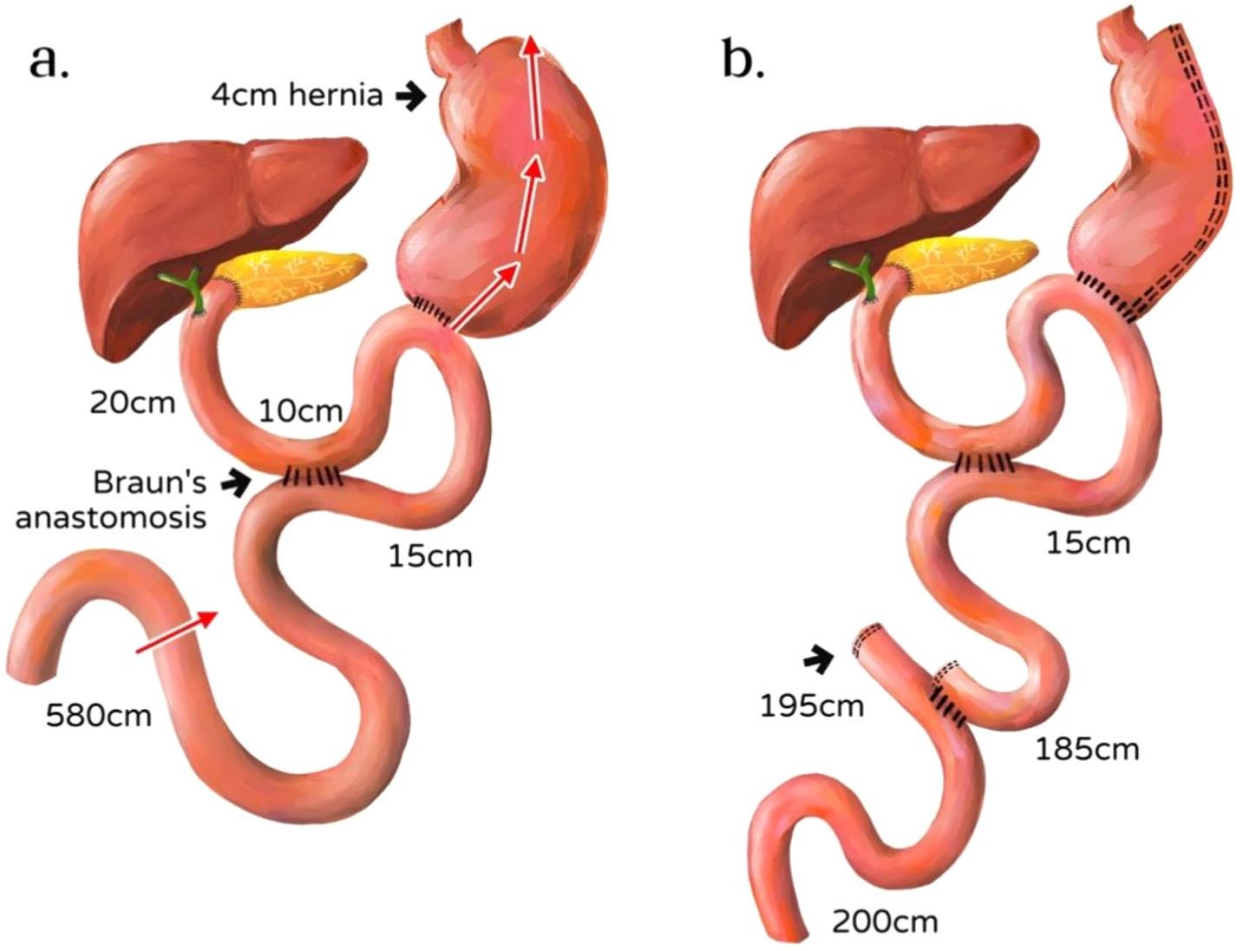
Schematic illustration of the laparoscopic modified one anastomosis gastric bypass (mOAGB) procedure in a patient with prior pancreaticoduodenectomy. (a) Preoperative anatomical mapping showing the status post‐conventional Whipple operation with a Braun enteroenterostomy. The solid red arrows indicate the planned resection lines: A sleeve gastrectomy initiated just distal to the existing gastrojejunostomy, and transection of the efferent jejunal limb 185 cm distal to the Braun anastomosis. Key bowel limb lengths are measured as shown. (b) Final postoperative anatomy following mOAGB. The stomach has been converted into a sleeve. A new side‐to‐side jejuno‐jejunostomy was created, bypassing a significant portion of the small bowel. This reconstruction resulted in a 195 cm excluded limb, a 185 cm jejunal limb, and a 200 cm ileal limb (400 cm common channel) for nutrient absorption. (Solid red arrows indicate cutting/stapling lines; dashed double lines indicate anastomoses or completed staple lines.)

The bariatric procedure consisted of two components. First, a vertical gastrectomy was performed to reduce gastric volume. The resection was initiated on the greater curvature near the existing GJ anastomosis and continued cephalad toward the angle of His using a 32‐French orogastric tube for calibration. This created a narrow restrictive gastric tube while preserving full patency of the original GJ outlet. Second, to introduce a bypass‐related metabolic component, we performed the “shortening efferent limb” step. The total intestinal length was measured to preserve a final common channel of 400 cm (from the GJ anastomosis to the ileocecal valve) in order to balance weight loss efficacy with nutritional safety. Accordingly, the efferent jejunal limb was transected approximately 185 cm distal to the original Braun anastomosis, and a side‐to‐side jejunojejunostomy was created, thereby bypassing an approximately 195‐cm proximal jejunal segment (Figure 1B).

The operation was completed laparoscopically in 181 min. Estimated blood loss was 50 mL. The postoperative course was uneventful; the patient tolerated a liquid diet on postoperative Day 1 and was discharged on Day 5. At the 1‐year follow‐up, the clinical outcome was excellent. The patient's weight decreased to 68.4 kg, corresponding to a total weight loss (%TWL) of 39.6% and a current BMI of 25.7 kg/m^2^. Her diabetes and hyperlipidemia reached complete remission, and hypertension improved significantly. Nutritional parameters remained stable with standard supplementation of multivitamins and iron. (Table 1).

**TABLE 1:** Longitudinal metabolic and nutritional profiles and perioperative outcomes and weight loss trajectory over 1 year.

Parameter	Pre‐operative	Post‐op 1 month	Post‐op 3 month	Post‐op 6 month	Post‐op 1 year	Reference range
*Metabolic control*						
HbA1c (%)	6.5	6	5.2	5.3	5.3	4.0–6.0
Glucose AC (mg/dL)	132	108	104	93	93	70–100
T‐Cholesterol (mg/dL)	172	155	161	177	174	< 200
Triglyceride (mg/dL)	167	204	146	91	96	< 150
*Nutritional status*						
Hemoglobin (g/dL)	12.3	—	—	—	11.9	12.0–16.0
Vitamin B12 (pg/mL)	—	—	—	—	124^a^	211–911
Folic acid (ng/mL)	—	—	—	—	4.1	> 5.38
*Organ function*						
Creatinine (mg/dL)	0.5	—	—	—	0.6	0.5–1.3
GOT (AST) (U/L)	25	—	32	—	11	< 35
GPT (ALT) (U/L)	24	—	25	—	—	< 35
*Weight loss trajectory*						
BMI (kg/m^2^)	42.6	33.2	29.6	28.2	25.7	
%TWL	—	21.9	30.4	33.8	39.6	
Clinical course and symptoms	Erosive esophagitis (grade A)	No dysphagia; No leak	No diarrhea; No dumping	Stable condition; No steatorrhea	No dumping	

*Note:* (—) indicates data not collected at that specific time point.

Abbreviations: %TWL, percentage of total weight loss; AC, ante cibum (fasting); BMI, body mass index; GOT, glutamic oxaloacetic transaminase; GPT, glutamic pyruvic transaminase; HbA1c, glycated hemoglobin; HHR, hiatal hernia repair; mOAGB, modified one anastomosis gastric bypass; T‐Cholesterol, total cholesterol.

^a^ Indicates value below the standard reference range, highlighting the need for vitamin supplementation.

Bariatric surgery in patients with post‐Whipple anatomy has rarely been reported and requires a delicate balance between metabolic efficacy and anatomical safety. To the best of our knowledge, this is the first reported case utilizing a modified OAGB‐like strategy for the management of severe obesity following PD [1, 2, 3]. This approach offers a distinct advantage by accepting the preexisting anatomy rather than attempting to dismantle it. By avoiding dissection around the pancreaticojejunostomy and choledochojejunostomy, the risk of life‐threatening biliopancreatic leakage may be substantially reduced. In the present case, the term mOAGB was used to reflect the conceptual similarity between this tailored reconstruction and the functional principles of OAGB, rather than to imply complete anatomical equivalence to a conventional primary OAGB. After sleeve gastrectomy, the post‐Whipple stomach became a long, narrow gastric conduit without a pylorus, functionally resembling the gastric pouch of OAGB. In addition, because the patient already had a GJ from the prior PD and an additional distal jejunojejunostomy was created to introduce a bypassed intestinal segment, the final configuration shared both restrictive and intestinal bypass features with OAGB. Nevertheless, this reconstruction should be interpreted as a tailored OAGB‐like procedure in altered post‐PD anatomy rather than a standard OAGB in the conventional bariatric sense.

A critical consideration in this specific population is the prevention of malnutrition. Patients post‐PD often have varying degrees of pancreatic exocrine insufficiency. Standard distal bypass procedures could exacerbate malabsorption, leading to severe protein‐calorie malnutrition or intractable steatorrhea. Therefore, our strategic decision to maintain a 400 cm common channel was paramount. Recent studies on OAGB have demonstrated that preserving a common channel length of at least 400 cm is critical to minimizing the risk of postoperative nutritional deficiencies without compromising weight loss efficacy [4, 5]. In a post‐Whipple patient with potential subclinical pancreatic exocrine insufficiency, adhering to this “400 cm safety rule” is vital to prevent catastrophic malnutrition.

Our 1‐year follow‐up data validates this tailored approach but also highlights the need for vigilance. The patient achieved excellent weight loss (39.6% TWL) and metabolic remission without signs of hypoalbuminemia, suggesting that the 400 cm channel successfully prevented protein malnutrition. However, the observed decline in Vitamin B12 levels (124 pg/mL) underscores the fragility of these patients. Even with a conservative bowel layout, the altered gastric acidity and bypass component can compromise micronutrient absorption. This finding emphasizes that lifelong nutritional surveillance and aggressive supplementation are nonnegotiable in post‐Whipple bariatric patients.

Regarding dumping syndrome, the loss of the pyloric sphincter combined with the sleeve gastrectomy presents a theoretical risk for rapid gastric emptying [4]. While our patient did not report severe clinical symptoms of dumping currently, this favorable outcome may be attributed to intestinal adaptation and volume restriction. Having undergone PD 5 years prior, the patient's efferent limb had likely adapted to tolerate faster gastric emptying [1]. Furthermore, the restrictive nature of the sleeve gastrectomy strictly limits the bolus size, preventing the rapid ingestion of large osmotic loads that typically trigger severe symptoms. As dumping syndrome is fundamentally volume‐dependent [1, 3, 4, 5], limiting the intake volume serves as a safeguard. Nevertheless, long‐term observation and continuous dietary counseling remain a cornerstone of postoperative care for these high‐risk individuals.

In conclusion, mOAGB with a 400 cm common channel represents a safe, logical, and effective option for managing severe obesity in patients with previous PD. It allows surgeons to intervene metabolically without navigating the hazardous zones of previous reconstruction. Our results suggest that while this configuration is effective for weight loss, it requires strict monitoring of micronutrients to ensure long‐term safety.

## Ethics Statement

All procedures performed in studies involving human participants were in accordance with the ethical standards of the institutional and/or national research committee and with the 1964 Helsinki declaration and its later amendments or comparable ethical standards.

## Conflicts of Interest

The authors declare no conflicts of interest.

## Data Availability

The data that support the findings of this study are available on request from the corresponding author. The data are not publicly available due to privacy or ethical restrictions.
